# Mitochondria and Neurodegenerative Diseases: A New Hotspot

**DOI:** 10.14336/AD.2022.1213

**Published:** 2023-08-01

**Authors:** Ang Li, Shuqin Cao, Kunlin Jin, Huanxing Su

**Affiliations:** ^1^State Key Laboratory of Quality Research in Chinese Medicine, Institute of Chinese Medical Sciences, University of Macau, Macao, China.; ^2^Department of Clinical Molecular Biology, University of Oslo and Akershus University Hospital, Lørenskog, Norway.; ^3^Clinical Biochemistry and Molecular Medicine, Department of Clinical Chemistry, Faculty of Allied Health Sciences, Chulalongkorn University, Bangkok, Thailand.; ^4^Department of Pharmacology and Neuroscience, University of North Texas Health Science Center, Fort Worth, TX 76107, USA

**Keywords:** Mitochondria, neurodegenerative diseases, drug development

## Abstract

Growing evidence suggests that the prevalence of neurodegenerative diseases (NDs) is on the rise with the aged population with substantially overlapping clinical and pathological features. The journal "Aging & Disease" portals are always responsive to publishing cutting-edge research on age-related neurodegeneration. Even though outstanding progress has recently been made in understanding NDs, the underlying mechanisms involved in neuronal degeneration are yet to be deciphered and addressed. There is credible evidence showing multiple links between mitochondria and NDs, gradually becoming the hotspot in mechanistic or drug development research. The editorial aims to reflect on and discuss some interesting and unique results from the papers published in "Aging & Disease" during the past three years (2020 - 2022).

Aging, the process of becoming older, is an inevitable physiological phenomenon, accompanied by many unexpected diseases such as cancer, cardiovascular disease, osteoporosis, and neurodegenerative diseases (NDs). Along with other causative factors like genetic mutations, aging has been considered a primary risk factor for most irreversible NDs [1]. Additionally, damaged mitochondria have been found in the aged with neurodegeneration like Alzheimer's disease (AD), Parkinson's (PD), Huntington's disease (HD), and Amyotrophic lateral sclerosis (ALS). Over the past few decades, researchers have become increasingly interested in mitochondrial functions, and several studies have focused on the mitochondrial changes associated with aging and disease in order to investigate the underlying pathogenic mechanisms and propose novel therapeutic approaches that extend healthy lifespans and/or provide cures for these debilitating conditions.

Mitochondria, an integral organelle of cells, play a vital role in cellular development, energy metabolic processes as well as clearance services, which further interact with cell aging and death. In health conditions, mitochondria are subject to strict regulatory processes to maintain the balance between mitochondrial biogenesis, mitochondrial dynamics, and mitophagy (specific autophagy to degrade damaged mitochondria) [2]. Impaired mitochondrial functions cause problems like energy shortage and cellular free radices accumulation, further contributing to the onset and progression of NDs such as AD, PD, HD, and ALS. This editorial commentary summarized the representative studies about the involvement of mitochondria in the development of NDs published in this journal, including the mitochondrial relevant pathogenic mechanism and the protective mechanism of potential drugs on improving mitochondrial function, which aims to point out the relevant phenotypes and drug development targets and providing ideas for future basic research and clinical treatment.

AD, the most common ND characterized by progressive cognitive impairments, is the most heavily invested and deeply studied. However, the mechanisms are still not fully uncovered, and no cure is available. The pathological development of AD includes brain atrophy, amyloid plaques (deposition of extracellular Aβ peptide aggregates), neurofibrillary tangles (accumulation of intracellular misfolded tau proteins), loss of neurons and synapses, dystrophic neurites, and dysfunctional mitochondria [3]. In addition, abnormal morphology and proliferation of astrocytes and microglia-related reactive gliosis have been reported in AD histopathological studies that indicate AD is a systematically degenerative disease, and various cells are involved [4].

Artemisinin (ART) is a very effective anti-malarial drug used for decades, saving millions of malaria patients worldwide. In 2020, Zhao et al. found ART benefits in alleviating the deposition of amyloid protein and tau protein, reducing the release of inflammatory and apoptotic factors, and inhibiting the death of neuronal cells in the AD mice model via the ERK/CREB signaling pathway [5]. Additionally, the authors confirmed that ART improves mitochondrial function and reduces oxidative stress *in vitro* and *in vivo* models. As a clinically widely used first-line drug, the advantage of ART is that it is worthwhile to validate its neuroprotective impact further and develop its therapeutic potential in AD without fear of noticeable hazardous side effects. This article provides an overview of how anti-AD drug candidates can protect neurons. However, further evidence is needed to prove the efficacy of neuronal protection.

In 2021, Adlimoghaddam et al. focused on mitochondrial dysfunction within astrocytes [6] and reported that the expression level of mitochondrial function-related proteins significantly changed in the 3xTg-AD mice astrocytes. Based on that, mitochondrial bioenergetics may be considered a new target for AD treatment. Nilotinib (Tasigna®, AMN107), a c-Abl tyrosine kinase inhibitor approved by the Food and Drug Administration (FDA) for patients with chronic myeloid leukemia (CML), was found to improve mitochondrial function (NF-κB signaling), mitochondrial dynamics and mitochondrial biogenesis parameters (CaMKII-PGC1α-Nrf2 pathway) of AD astrocytes and play an obvious protective effect. Although astrocytes display morphological alterations in AD that have been known for over a century, neuronal-based research is still the focus of neurodegenerative studies. The study of Adlimoghaddam et al. shows that astroglia could be a therapeutic target in AD. Meanwhile, maintaining mitochondria function and targeting mitochondrial bioenergetics might be another promising strategy for AD treatment. Additionally, reprogramming apoptotic pathways in cancer cells is associated with mitochondria and metabolic pathways. Thus, Nilotinib, a drug used to treat cancer, can serve as a model for the screening and possible repurposing of current anti-cancer therapies as bi-functional drugs to treat AD, and this might speed up research for treatments for AD.

Mitochondrial dysfunction and impaired mitophagy are believed to speed up AD progression. In 2022, Wang et al. summarized how mitophagy is linked to AD, the currently known AD models, and mitophagy inducers, which turn up mitophagy through small molecule compounds like MNM (NAD^+^ precursor), UA, and AC supplementation. In APP/PS1 and 3xTg-AD mice, these compounds could further increase the microglia's phagocytic capacity and inhibit neuroinflammation by activating mitophagy, thus alleviating AD progression [7]. Furthermore, the systematic review of mitophagy inducers in clinical trials helps us better understand cutting-edge drug research and development progress. Mitophagy inducer treatment is feasible but checking whether the mitophagy-inducing candidates cause mitochondrial toxicity is required. Previously, most known mitophagy inducers (Carbonyl cyanide 3-chlorophenylhydrazone or Carbonyl cyanide 4-(trifluoromethoxy)phenylhydrazone) induce mitophagy *via* damaging mitochondria, which are not well-matched drug candidates. Therefore, multi-drug screening models are recommended, like a combination of artificial intelligence and wet-lab validation, as mentioned in this review.

Accumulation of misfolded protein is considered a pathological feature of NDs. A dysfunctional clearance system that may induce and/or accelerate the progression of the disease process has been gradually recognized in NDs. Repairing the intracellular clearance system has become a new research focus. Mitophagy is garnering increasing interest from experts involved in AD and PD research. For example, TFEB (transcription factor EB), a key regulator of lysosomal biogenesis and autophagy, plays a pivotal role in the degradation of protein aggregates and damaged organelles and has thus been regarded as a promising therapeutic target for NDs [8].

PD, the second most common ND, is characterized by the progressive loss of dopaminergic neurons and the accumulation of α-synuclein, a major component of Lewy bodies in the substantia nigra. In 2022, Yang et al. discussed TFEB regulation in AD and PD and systematically summarized the protective small-molecule TFEB activators in animal models of NDs, showing great potential for further development as novel anti-neurodegenerative drugs [9]. Importantly, the specific TFEB activators are still lacking, and most of the reported TFEB activators work by inhibiting mTOR activity, which plays a major role in regulating normal neuronal function, further demonstrating the relationship between them and the pros and cons that deserves further investigation. Even the autophagolysosomal pathway might be considered one of the best options for removing accumulated aggregates and damaged organelles. The therapeutic candidate may be preferable if it can activate TFEB safely and effectively, facilitating the clearance of aberrant protein aggregation and/or damaged organelles like mitochondria. Long-term autophagy/mitophagy maintenance may have important implications for neuronal cells; however, this must be considered and verified.

HD, an adult-onset ND is caused by a trinucleotide CAG repeat expansion in the HTT gene. In 2021, Machiela et al. utilized the transgenic *C. elegans* models of HD and found the disease phenotype of mitochondrial fragmentation in the pathogenesis of HD, which is temporally correlated with polyglutamine aggregation [10]. Additionally, abnormally expressed mitochondrial fission and fusion genes have been noticed in HD worms. Dynamin-related protein 1 (*Drp1*) and fission protein 1 (*Fis1*) play a vital role in mitochondrial fission regulation, which is recruited to the outer mitochondrial membrane where the latter causes a constriction and thereby initiates fission. Interestingly, the authors found no correlation between reduced *Drp1* expression level and defective mitochondria, but toxicity was reported. Also, three target genes with mitochondrial morphology protective effects in HD worms are reported from the RNAi knockdown examination. This paper exemplifies the use of the *C. elegans* model for HD research by explaining the function of mitochondrial fragmentation in HD and identifying prospective intervention targets that might aid in the development of future treatments. More advanced animal models, such as mice, are still needed for further verification of the effectiveness of the specified target. Fortuitously, many genes are conserved across species, making future research possible.

ALS is a rare progressively motor neuron (MN) disease affecting people of all races and ethnic backgrounds [11]. Radicava, a free radical scavenger, is thought to be able to alleviate the effects of oxidative stress, thereby slowing the progression of ALS, and was approved by the FDA for the clinical treatment of ALS in 2017. Although several in-depth research on this topic has been conducted, the role of mitochondria in ALS has not been systematically elucidated. Likewise, it is unclear if or whether mitochondria are a target for the development of ALS therapies and the relevance of dysfunctional mitochondria and impaired mitophagy play in the disease. Likewise, it is crucial to investigate glial cells, particularly microglia and astrocytes, in addition to motor neurons in ALS research. All of these lacunas in understanding mitochondria and ALS warrant further investigation.

Despite years of human endeavour, no therapeutic breakthrough in treating NDs has been achieved. The association between mitochondria and NDs has recently emerged as a new hot spot. Numerous small natural compounds or plants homologous to medicine and food have been recorded as possessing protective effects on NDs through the intervention of mitochondria. In 2022, Zaman et al. summarized the plant-derived astragaloside IV's role in treating neuronal aging by suppressing oxidative stress, attenuating inflammatory responses, and maintaining mitochondrial integrity [12]. In 2022, Balakrishnan et al. summarized that black pepper and its bioactive compounds, especially piperine have obvious neuroprotective effects in age-related neurological disorders by maintaining mitochondrial function, promoting mitochondrial biogenesis and resisting oxidative stress [13]. Overall, a hope for a potential cure for NDs may be bolstered by an influx of new research and promising medication discoveries.
